# Prognostic prediction and multidimensional dissections of a macrophages M0-related gene signature in liver cancer

**DOI:** 10.3389/fendo.2023.1153562

**Published:** 2023-03-24

**Authors:** Xiaoming Xu, Jingzhi Wang

**Affiliations:** ^1^ Department of Gastroenterology, Jining First People’s Hospital, Jining, China; ^2^ Department of Radiotherapy Oncology, The Affiliated Yancheng First Hospital of Nanjing University Medical School, The First People’s Hospital of Yancheng, Yancheng, China

**Keywords:** liver cancer, macrophages M0, prognostic signature, immune infiltration, immunotherapy, single-cell analysis

## Abstract

**Background:**

Liver hepatocellular carcinoma (LIHC) is the seventh most commonly diagnosed malignancy and the third leading cause of all cancer death worldwide. The undifferentiated macrophages M0 can be induced into polarized M1 and M2 to exert opposite effects in tumor microenvironment. However, the prognostic value of macrophages M0 phenotype remains obscure in LIHC.

**Methods:**

The transcriptome data of LIHC was obtained from TCGA database and ICGC database. 365 LIHC samples from TCGA database and 231 LIHC samples from ICGC database were finally included. Macrophages M0-related genes (MRGs) were screened by Pearson correlation analysis and univariate Cox regression analysis based on the infiltration level of Macrophages M0. LASSO regression analysis was employed to construct a prognostic signature based on MRGs, and risk scores were accordingly calculated. Then we investigated the MRGs-based prognostic signature with respects to prognostic value, clinical significance, strengthened pathways, immune infiltration, gene mutation and drug sensitivity. Furthermore, the expression pattern of MRGs in the tumor microenvironment were also detected in LIHC.

**Results:**

A ten-MRG signature was developed and clarified as independent prognostic predictors in LIHC. The risk score-based nomogram showed favorable capability in survival prediction. Several substance metabolism activities like fatty acid/amino acid metabolism were strengthened in low-risk group. Low risk group was deciphered to harbor TTN mutation-driven tumorigenesis, while TP53 mutation was dominant in high-risk group. We also ascertained that the infiltration levels of immune cells and expressions of immune checkpoints are significantly influenced by the risk score. Besides, we implied that patients in low-risk group may be more sensitive to several anti-cancer drugs. What’s more important, single-cell analysis verified the expression of MRGs in the tumor microenvironment of LIHC.

**Conclusion:**

Multidimensional evaluations verified the clinical utility of the macrophages M0-related gene signature to predict prognosis, assist risk decision and guide treatment strategy for patients with LIHC.

## 1 Introduction

According to GLOBOCAN statistics 2020, liver cancer is reported to be the seventh most commonly diagnosed malignancy, with over 900,000 new cases per year, while it is the third leading cause of all cancer death (8.3%), which induces a huge disease burden worldwide (1). Liver hepatocellular carcinoma (LIHC) and intrahepatic cholangiocarcinoma (ICC) are the two major histopathological subtypes of liver cancer in clinics, accounting for over 90% of cases (1). Currently, surgical resection is still the primary therapy strategy for liver cancer, and other treatments, including interventional therapy, chemo/radiotherapy, molecular targeted therapy, and immunotherapy are considered supplementary methods. With the development of comprehensive treatment, the prognosis of patients with liver cancer has been partially prolonged (2, 3). However, the whole prognosis of liver cancer remains unsatisfactory on account of concealed early symptoms, local recurrence, and distant metastasis (4). TNM stage is the traditional method to assess the prognosis of patients, whereas it has particular limitations, for it can only analyze the clinical outcome at a macro level. In the era of precision medicine, it is prevalent to process prognostic evaluation utilizing a comprehensive molecular signature, especially in cancer studies. Therefore, the identification of a reliable gene signature to predict the prognosis of patients with liver cancer may contribute to clinical management and risk decision, rendering possible priority for survival improvement.

Tumor microenvironment (TME) is a sophisticated ecosystem that ameliorates tumor growth by promoting angiogenesis and supporting immunosuppression (5). Notably, the interactive mechanisms between cancer cells and diverse immune infiltrating cells have been increasingly focused, in an attempt to exploit novel anticancer strategies. The facts suggest that immune infiltrating cells may exert tumor-promoting effects by driving chronic inflammation and blinding host immune surveillance (5). Commonly, danger-associated molecular patterns (DAMPs) and pathogen-associated molecular patterns (PAMPs) as stimuli to stir tissue homeostasis can be identified by pattern recognition receptors on the surface of innate immune cells like neutrophils, macrophages, dendritic cells, and NK cells, thereby subsequently inflaming the TME (6, 7). However, the inflammation remains unlocked and becomes chronic in TME, which significantly benefits cancer cells (8). Multiple factors gradually remodel the ECM toward more tumor-friendly (8, 9). Macrophages, have been determined to propel tumor progression by enhancing angiogenesis, invasion, and metastasis *in vivo* according to their functional status induced by the TME (10). It is believed that the diversity of macrophages can be employed by cancer cells to contribute to progression utilizing EGF stimulation, for instance (10). Considering the pivotal roles of macrophages in cancer development, previous studies managed to establish favorable prognostic models utilizing macrophages-related genes (MRGs) in several malignancies (11–13). However, the prognostic value of MRGs in liver cancer remains obscure.

Liver cancer cells express PDL1 to inhibit the activity of cytotoxic T cells, so as to evade immune surveillance and infinitely proliferate. Immunocheckpoint inhibitors can reverse the inhibition of liver cancer cells on cytotoxic T cells, rendering cytotoxic T cells active to kill cancer cells (14). At present, immunotherapy for LIHC presents a multi-plan situation. The combination of the anti-PDL1 antibody atezolizumab and the anti-angiogenesis antibody bevacizumab is getting standard in first-line therapy. The anti-PD1 antibody nivolumab and pembrolizumab can also be sequentially applied after tyrosine kinase inhibitor (TKI) in several conditions (15). The current bottlenecks for immunotherapy of LIHC are the exploitation of novel predictive tools to assess therapeutic efficacy and conducting clinical trials to widen the applicable patients, as well as discovering more effective dosage regimens (16).

**Figure 1:**
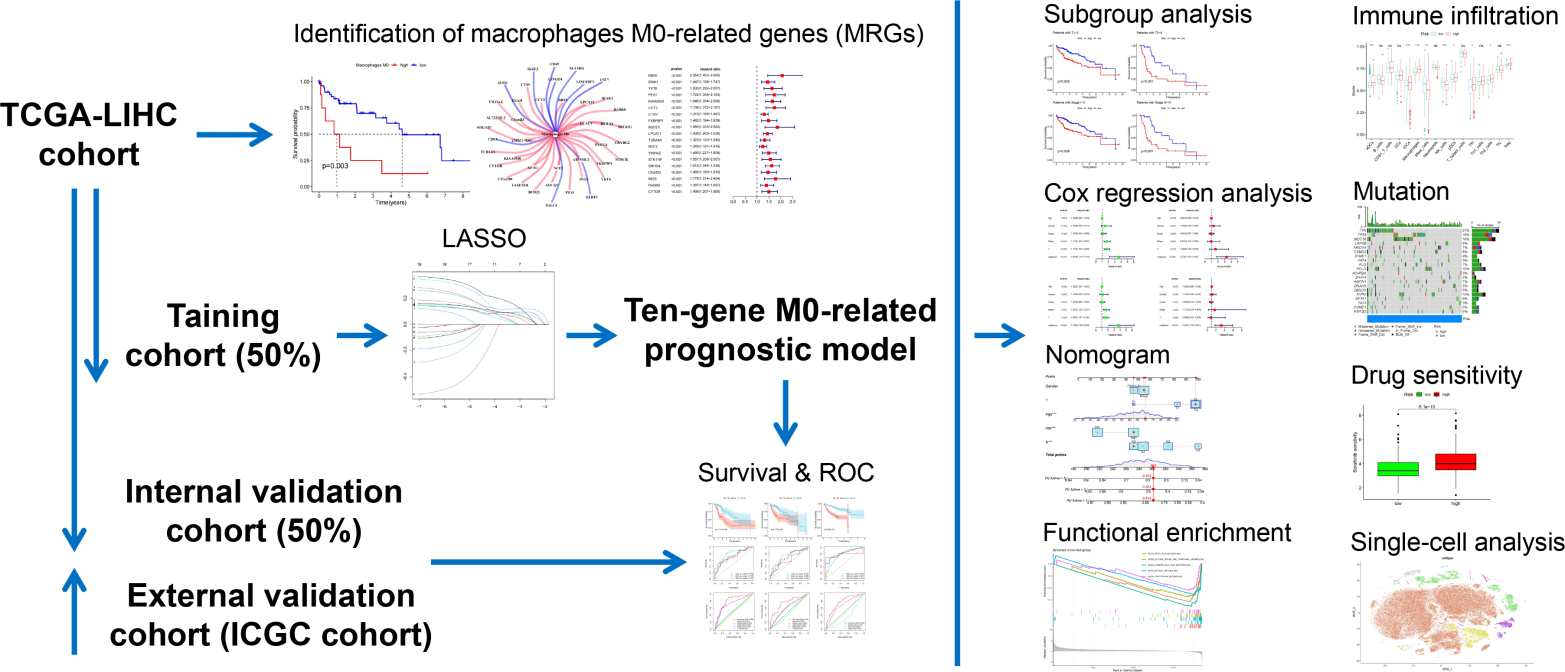
Workflow of the present study.

In this present study, we managed to develop and validate a prognostic signature based on MRGs, through which better risk decisions may be achieved in clinics. Distinct subgroups were also classified based on MRGs. Investigations of the gene signature concerning clinical subgroup, functional characterization, immune infiltration, immune checkpoint expression, and mutation landscape were organized. We also provided implications of drug agents *via* IC50 drug sensitivity analysis. Moreover, single-cell analysis determined the expression pattern of MRGs in the TME of LIHC. The workflow of the present study is summarized in **Figure 1**.

## 2 Materials and methods

### 2.1 Data acquisition and preprocessing

RNA-sequencing data and clinical information of liver hepatocellular carcinoma (LIHC) samples were downloaded from the TCGA database (http://cancergenome.nih.gov) and the ICGC database (https://dcc.icgc.org). Samples without complete survival information were excluded. We thus finally enrolled 365 LIHC samples from TCGA database and 231 LIHC samples from ICGC database.

### 2.2 Identification of M0-related genes

Above all, we quantified the infiltration levels of 22 immune cells of each TCGA-LIHC sample by the CIBERSORT algorithm. Then the survival difference between the low infiltration group and the high infiltration group of a specific immune cell was investigated. Pearson correlation analysis was used to identify the genes (MRGs) significantly correlated with macrophages M0. Genes with |*r*| > 0.4 and *P* < 0.001 were considered significantly relevant. We next processed Gene Ontology (GO)/Kyoto Encyclopedia of Genes and Genomes (KEGG) functional enrichment analyses of MRGs based on cluster Profiler and org.Hs.eg.db R packages. Univariate analysis was conducted to further filter MRGs that harbor significant prognostic importance.

### 2.3 Consensus clustering of LIHC based on M0-related genes

Consensus clustering was employed to testify the consistency of selected MRGs by means of dissecting different LIHC subtypes in TCGA cohort. We compared the MRGs expressions and infiltration levels of immune cells between LIHC subtypes. Survival differences between the subtypes were also determined.

### 2.4 Construction and validation of the prognostic signature based on M0-related genes

TCGA cohort was randomly divided into the training cohort (50%) and internal validation cohort (50%) respectively, while the ICGC cohort was used as the external validation cohort. LASSO regression analysis was employed to construct a prognostic signature based on MRGs in the training cohort. Risk score = ∑(C_i_*E_i_), *i* represented a certain MRG, *C* represented the coefficient of MRG and *E* represented the expression level of MRG. The low-risk group and high-risk group were evenly divided according to the median cut-off value of the risk score. Principal component analysis (PCA) was utilized to check out the discrimination between the high-risk group and the low-risk group. We also compared the survival difference between the low-risk group and the high-risk group *via* survminer and survival R packages. The predictive capability of the prognostic signature was tested by receiver operating characteristic (ROC) curves *via* the timeROC R package. Corresponding analyses were also performed in the internal validation cohort and the ICGC cohort.

### 2.5 Clinical attachment of the prognostic signature and establishment of nomogram

We applied the prognostic signature in several clinical subgroups to further determine its clinical prognostic utility. Next, univariate and multivariate Cox regression analyses were conducted to decipher independent prognostic predictors for LIHC from several clinicopathological parameters and risk score in both the TCGA cohort and the ICGC cohort. We subsequently developed a nomogram to predict overall survival (OS) utilizing several clinicopathological factors. The predictive accuracy of the nomogram was verified by calibration curves.

### 2.6 Functional strengthens of the two risk groups

The differentially expressed genes (DEGs) between the low-risk group and the high-risk group were identified with the DEGseq R package. Genes with |Log_2_FC| > 1 and *P* < 0.05 were considered DEGs. Gene Set Enrichment Analysis (GSEA) was performed to determine the significantly enriched functional characterizations in the two risk groups, respectively.

### 2.7 Differences of immune infiltration and immune checkpoint expression between the two risk groups

We compared the activity of several immune activities between the low-risk group and the high-risk group utilizing single sample gene set enrichment analysis (ssGSEA), as well as the infiltration levels of various immune cells. Moreover, we investigated the expression pattern of 40 immune checkpoints between the two risk groups to ascertain the potential value of the prognostic signature in immunotherapy.

### 2.8 Mutation landscapes of the two risk groups

The mutation landscapes of the low-risk group and the high-risk group were obtained *via* the maftools R package, respectively. The top twenty most frequently altered genes in the two risk groups were displayed respectively. The difference in tumor mutation burden (TMB) between the low-risk group and the high-risk group was checked out. Besides, low TMB group and high TMB group were divided according to the median cut-off value of TMB. Survival differences between patients in the low-TMB group and the high-TMB group with or without combination of risk groups were further uncovered.

### 2.9 Drug sensitivity analysis

With the pRRophetic R package, we processed broad drug screening based on the GDSC database (https://www.sanger.ac.uk/tool/gdsc-genomics-drug-sensitivity-cancer/) to ascertain the drug agents that the two risk groups may sensitively respond to.

### 2.10 Single-cell RNA-sequencing data analysis

About 110992 high-quality cells were filtered and obtained from the LIHC_GSE189903 dataset. The expression pattern of the MRGs were visualized by the Seurat R package based on the single-cell profile of LIHC_GSE189903.

### 2.11 Statistical analysis

Bioinformatic analyses were all conducted by R 4.0.3. The comparison of the K-M survival curve was achieved by Cox regression analysis. Differences in expression levels between groups were compared by the Wilcoxon rank sum test. Pearson correlation was taken for correlation analysis. *P* < 0.05 was deemed statistically significant. “*” indicates *P* < 0.05, “**” indicates *P* < 0.01 and “***” indicates *P* < 0.001 throughout this study.

## 3 Results

### 3.1 Macrophages M0 abundance extremely correlated with the prognosis of LIHC

**Figure 2:**
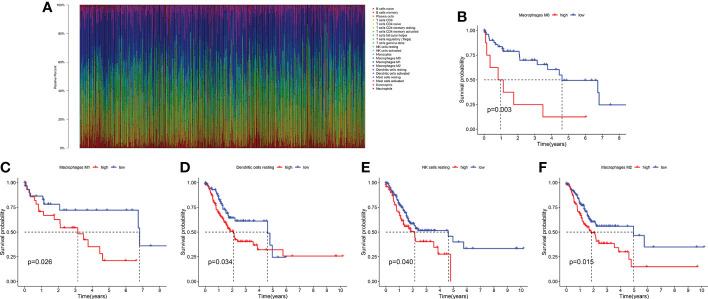
The survival significance of 22 immune infiltrating cells in LIHC. **(A)** Quantification of the infiltration levels of 22 immune cells in the TCGA cohort. **(B)** The survival significance of macrophages M0. **(C)** The survival significance of macrophages M1. **(D)** The survival significance of dendritic cells resting. **(E)** The survival significance of NK cells resting. **(F)** The survival significance of macrophages M2.

**Figure 3:**
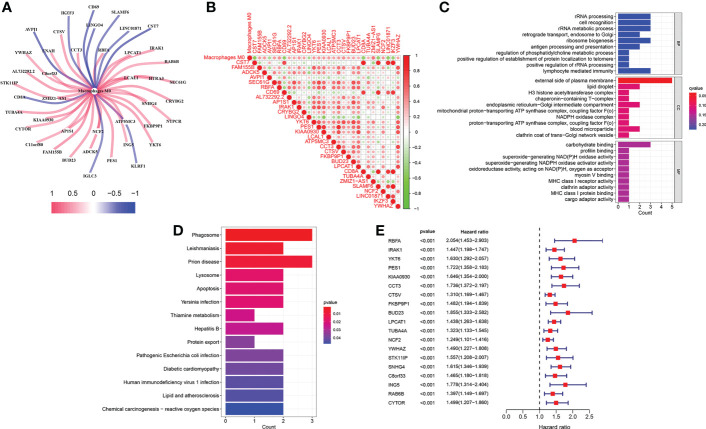
Identification of M0-related genes. **(A)** Correlations between MRGs and macrophages M0. **(B)** Correlations within the 31 MRGs. **(C)** GO functional enrichment analysis of the MRGs. **(D)** KEGG functional enrichment analysis of the MRGs. **(E)** Univariate Cox regression analysis of the MRGs.

The infiltration levels of 22 immune cells of each TCGA-LIHC sample were qualified (**Figure 2A**). We found that the survival difference between high- and low macrophages M0 infiltration groups is the most significant according to its polarized *P*-value (*P* = 0.003) among the 22 immune infiltrating cells (**Figures 2B–F**). Patients with higher infiltration levels of macrophages M0 suffered from poorer outcomes. A total of 31 MRGs were identified to be significantly correlated with macrophages M0 in LIHC, among which ten genes showed positive correlation and the other 21 genes showed negative correlation (**Figure 3A**). Most MRGs positively correlated with each other (**Figure 3B**). GO/KEGG functional enrichment analyses indicated that these MRGs are enriched in the external side of the plasma membrane, phagosome, lysosome, apoptosis, protein export, and chemical carcinogenesis-oxidative oxygen species, etc. (**Figures 3C, D**). Univariate Cox regression analysis further determined 19 MRGs significantly correlated with the prognosis of LIHC (*P* < 0.001) (**Figure 3E**).

### 3.2 Two subtypes were divided based on M0-related genes in LIHC

**Figure 4:**
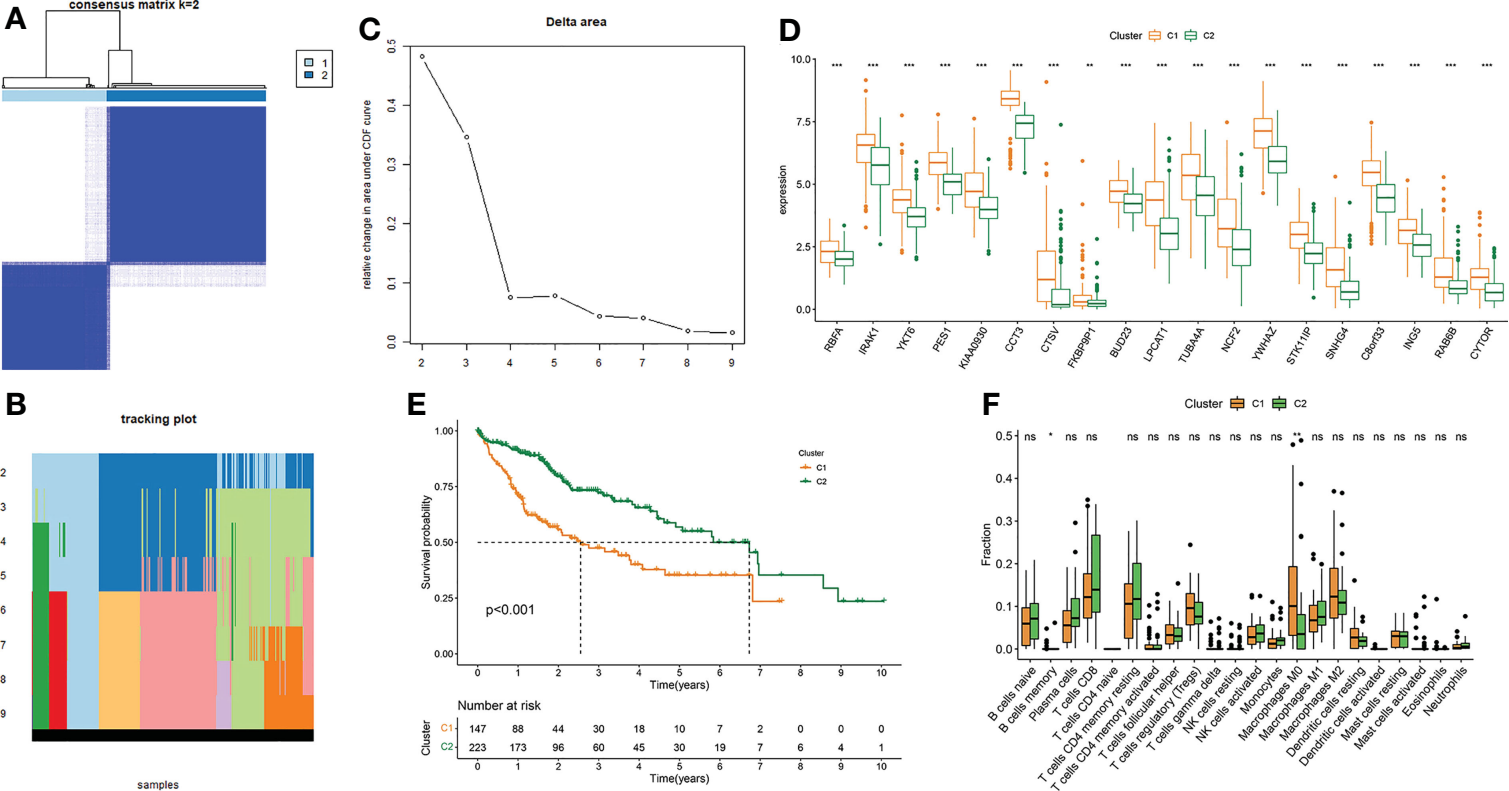
Consensus clustering of LIHC based on MRGs. **(A–C)** Consensus clustering. **(D)** Differential expressions of the 19 MRGs between subtype 1 and subtype 2. **(E)** Survival difference between subtype 1 and subtype 2. **(F)** Differences in 22 immune cells’ infiltration levels between subtype 1 and subtype 2. *P-value < 0.05, **P-value < 0.01 and ***P-value < 0.001, ns, represents non-significant.

We divided TCGA-LIHC samples into subtype 1 and subtype 2 based on 19 MRGs (**Figures 4A–C**). The 19 MRGs were all differentially expressed between the two subtypes (**Figure 4D**). Subtype 1 with higher infiltration levels of macrophages M0 harbored a worse prognosis than subtype 2 (*P* < 0.001) (**Figures 4E, F**).

### 3.3 A ten-gene signature was constructed and validated for prognosis prediction in LIHC

**Figure 5:**
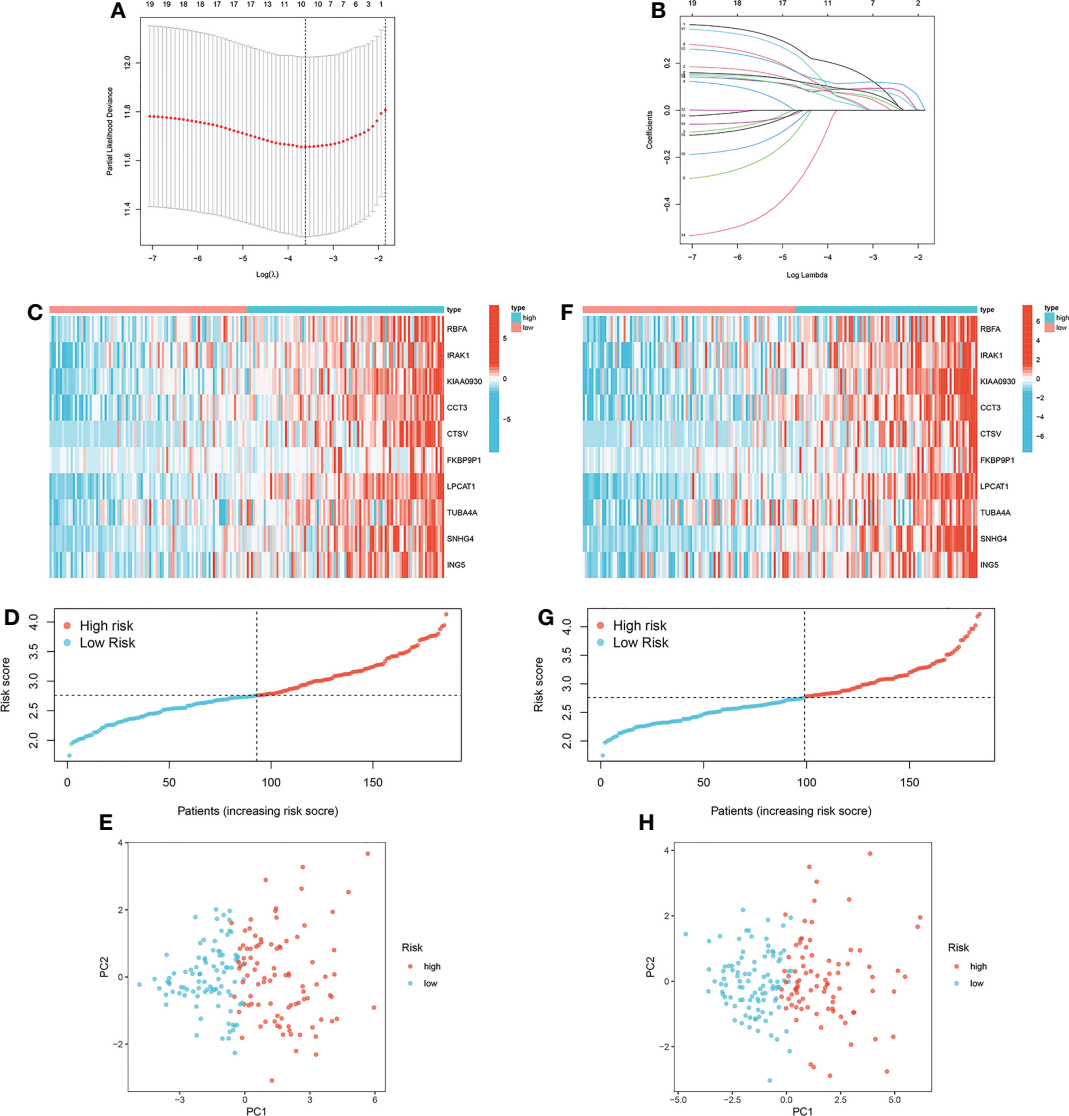
Construction of the prognostic signature based on MRGs. **(A, B)** LASSO regression analysis in the training cohort. **(C)** Expression pattern of the ten MRGs between the low-risk group and the high-risk group in the training cohort. **(D)** Distribution of patients with risk scores in different risk groups in the training cohort. **(E)** Principal component analysis in the training cohort. **(F)** Expression pattern of the ten MRGs between the low-risk group and the high-risk group in the internal validation cohort. **(G)** Distribution of patients with risk scores in different risk groups in the internal validation cohort. **(H)** Principal component analysis in the internal validation cohort.

A ten-gene signature was generated by LASSO regression analysis in the training cohort (**Figures 5A, B**). Risk score = 0.1308 * RBFA exp. + 0.0489 * IRAK1 exp. + 0.0882 * KIAA0930 exp. + 0.0936 * CCT3 exp. + 0.0735 * CTSV exp. + 0.1284 * FKBP9P1 exp. + 0.1209 * LPCAT1 exp. + 0.0873 * TUBA4A exp. + 0.058 * SNHG4 exp. + 0.075 * ING5 exp. The expression pattern of the ten MRGs between the low-risk group and the high-risk group was visualized (**Figure 5C**). The distribution of patients with risk scores in different risk groups was displayed (**Figure 5D**). PCA further verified the distinct demarcation between the low-risk group and the high-risk group (**Figure 5E**). Corresponding investigations were performed in the internal validation cohort (**Figures 5F–H**).

**Figure 6:**
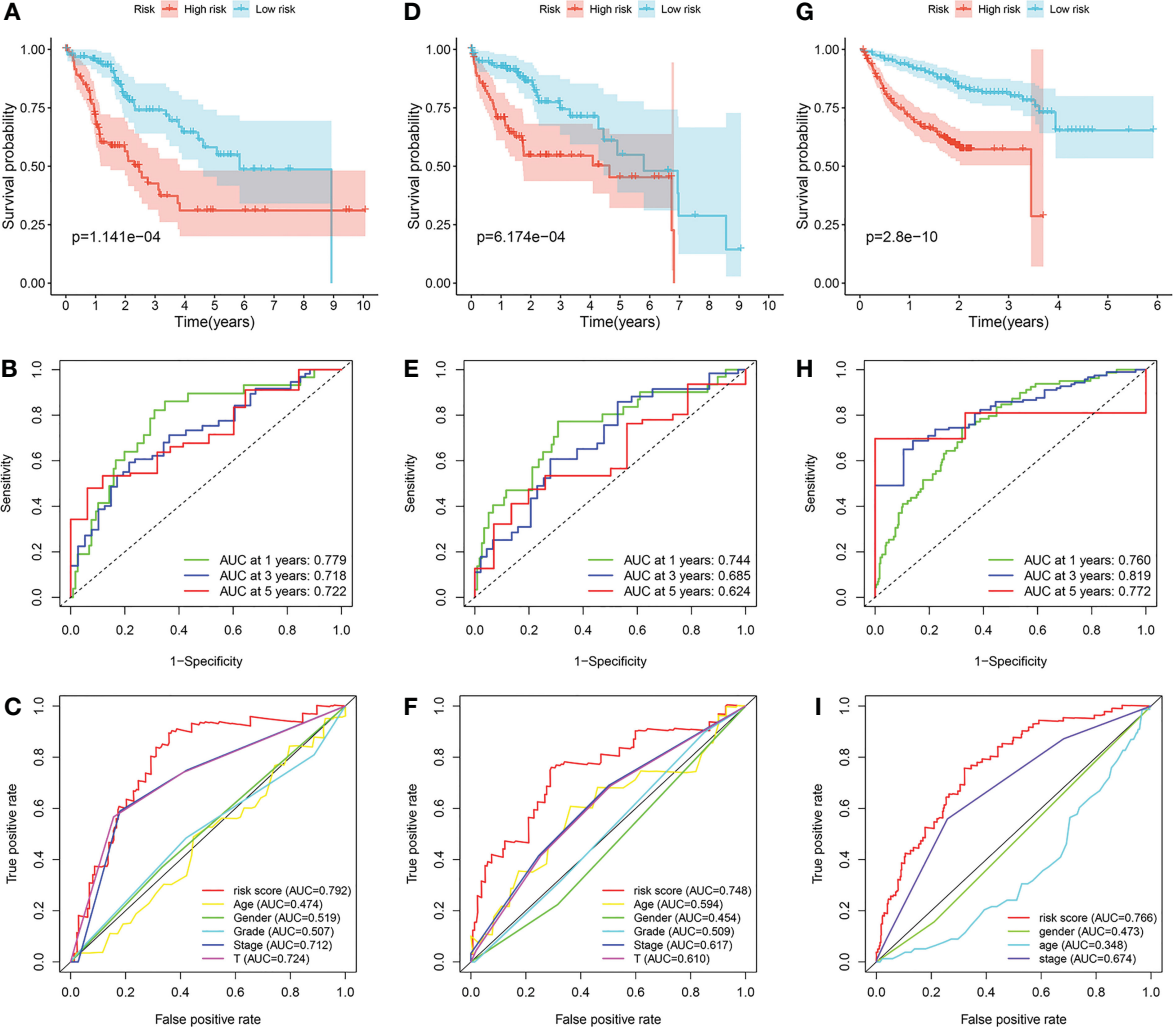
Validation of the prognostic signature. **(A–C)** Application of the prognostic signature in the training cohort. **(D–F)** Application of the prognostic signature in the internal validation cohort. **(G–I)** Application of the prognostic signature in the ICGC cohort.

The survival differences between the low-risk group and the high-risk group in the training cohort, internal validation cohort and ICGC cohort were all well distinguished (**Figures 6A, D, G** **)**. In the training cohort, the AUCs at 1-, 3- and 5-year were 0.779, 0.718, and 0.722 (**Figure 6B**). In the internal validation cohort, the AUCs at 1-, 3- and 5-year were 0.744, 0.685, and 0.624 (**Figure 6E**). In the ICGC cohort, the AUCs at 1-, 3- and 5-year were 0.760, 0.819, and 0.772 (**Figure 6H**). Furthermore, we found that the prediction capability of the prognostic signature is better than any other clinical characteristics, for its general AUCs were 0.792, 0.748, and 0.766 in the three cohorts respectively (**Figures 6C, F, I** **)**.

### 3.4 The risk score was identified as an independent prognostic predictor for LIHC

**Figure 7:**
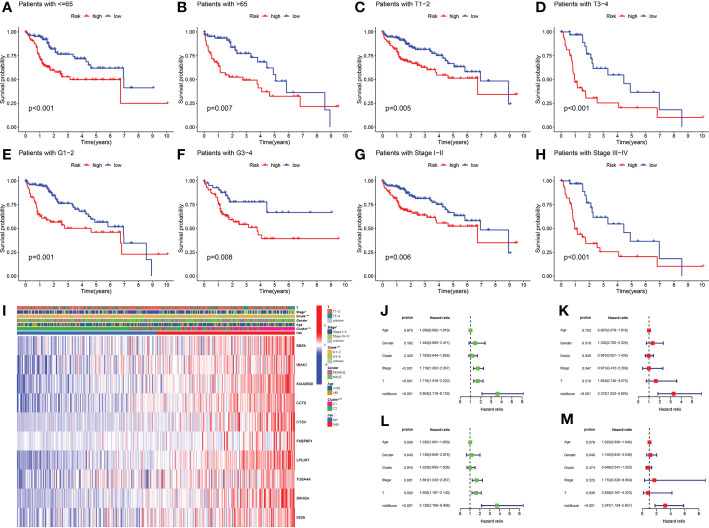
Clinical analyses of the prognostic signature. **(A)** LIHC patients with ≤ 65. **(B)** LIHC patients with > 65. **(C)** LIHC patients with T1-T2. **(D)** LIHC patients with T3-T4. **(E)** LIHC patients with G1-G2. **(F)** LIHC patients with G3-G4. **(G)** LIHC patients with stage I-II. **(H)** LIHC patients with stage III-IV. **(I)** Distributions of clinicopathological parameters between the low-risk group and the high-risk group. **(J)** Univariate Cox regression analysis of risk score in TCGA cohort. **(K)** Multivariate Cox regression analysis of risk score in TCGA cohort. **(L)** Univariate Cox regression analyses of risk score in ICGC cohort. **(M)** Multivariate Cox regression analysis of risk score in ICGC cohort. *P-value < 0.05, ***P-value < 0.001.

Firstly, we applied the prognostic signature in eight clinical subgroups (**Figures 7A–H**). Results confirmed the broad applicability of the prognostic signature in all types of patients with LIHC. Distributions of several clinical parameters between the low-risk group and the high-risk group were also demonstrated (**Figure 7I**). We next identified risk score as an independent prognostic predictor for LIHC in both the TCGA cohort and the ICGC cohort by Cox regression analyses, which verified the strong prognostic value of the prognostic signature (*P* < 0.001) (**Figures 7J–M**).

### 3.5 The risk score-based nomogram showed favorable prediction capability

**Figure 8:**
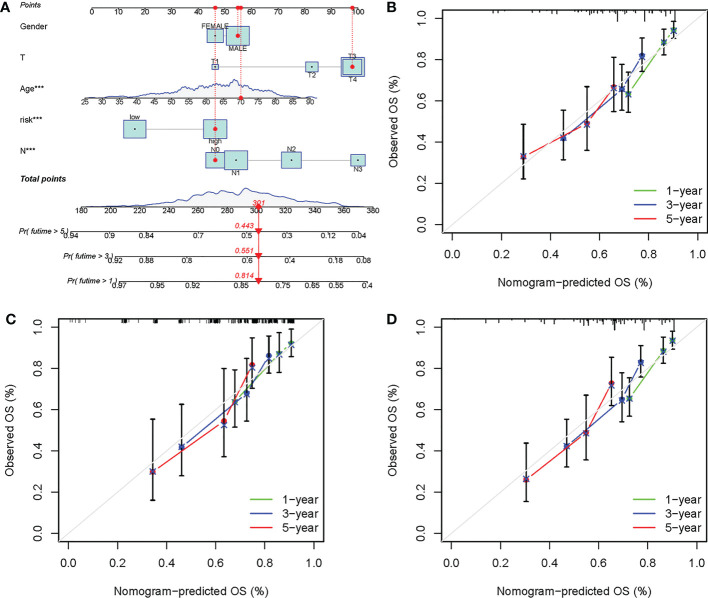
Development of a prognostic nomogram based on the risk score. **(A)** Development of the nomogram based on clinicopathological parameters and risk score in the training cohort. Calibration curves at 1-, 3- and 5-year in the **(B)** ICGC cohort, **(C)** training cohort, and **(D)** internal validation cohort. ***P-value < 0.001.

A nomogram was further developed based on several clinicopathological factors and risk score for OS prediction in the training cohort (**Figure 8A**). Calibration curves were employed to examine the predictive accuracy, which was close to the ideal line, suggesting excellent predictive efficacy of the nomogram in the ICGC cohort, training cohort, and internal validation cohort (**Figures 8B–D**).

### 3.6 Substance metabolism activities were strengthened in low-risk group

**Figure 9:**
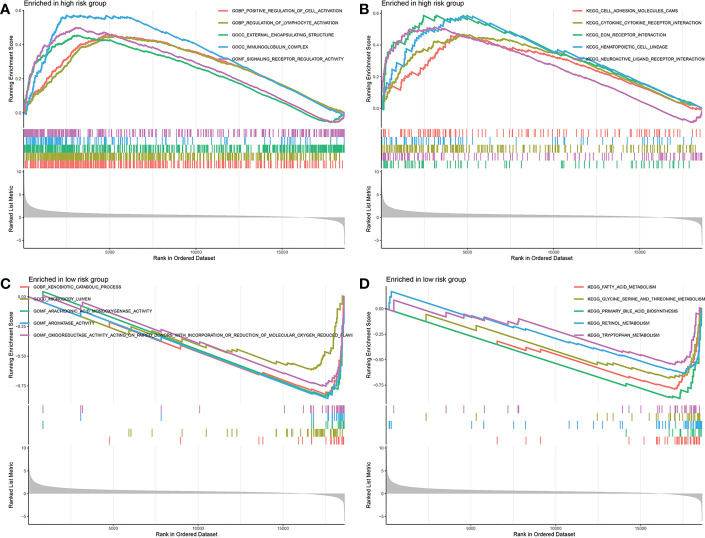
Gene set enrichment analysis. **(A, B)** Biological activities enriched in the high-risk group. **(C, D)** Biological activities enriched in the low-risk group.

Above all, the DEGs between the low-risk group and the high-risk group were ascertained. The DEGs in different risk groups were submitted to GSEA functional enrichment analysis, respectively. The biological activities that are significantly enriched in the high-risk group were positive regulation of cell activation, regulation of lymphocyte activation, external encapsulating structure, immunoglobulin complex, signaling receptor regulator activity, cell adhesion molecules cams, cytokine-cytokine receptor interaction, ECM receptor interaction, hematopoietic cell lineage, and neuroactive ligand receptor interaction (**Figures 9A, B**). The biological activities that are significantly enriched in the low-risk group were xenobiotic catabolic process, microbody lumen, arachidonic acid monooxygenase activity, aromatase activity, oxidoreductase activity acting on paired donors with incorporation, fatty acid metabolism, glycine serine and threonine metabolism, primary bile acid biosynthesis, retinol metabolism, and tryptophan metabolism (**Figures 9C, D**). It appeared to be that the substance metabolism activities are significantly strengthened in the low-risk group.

### 3.7 The risk score correlated with higher immune infiltration and immune checkpoint expression

**Figure 10:**
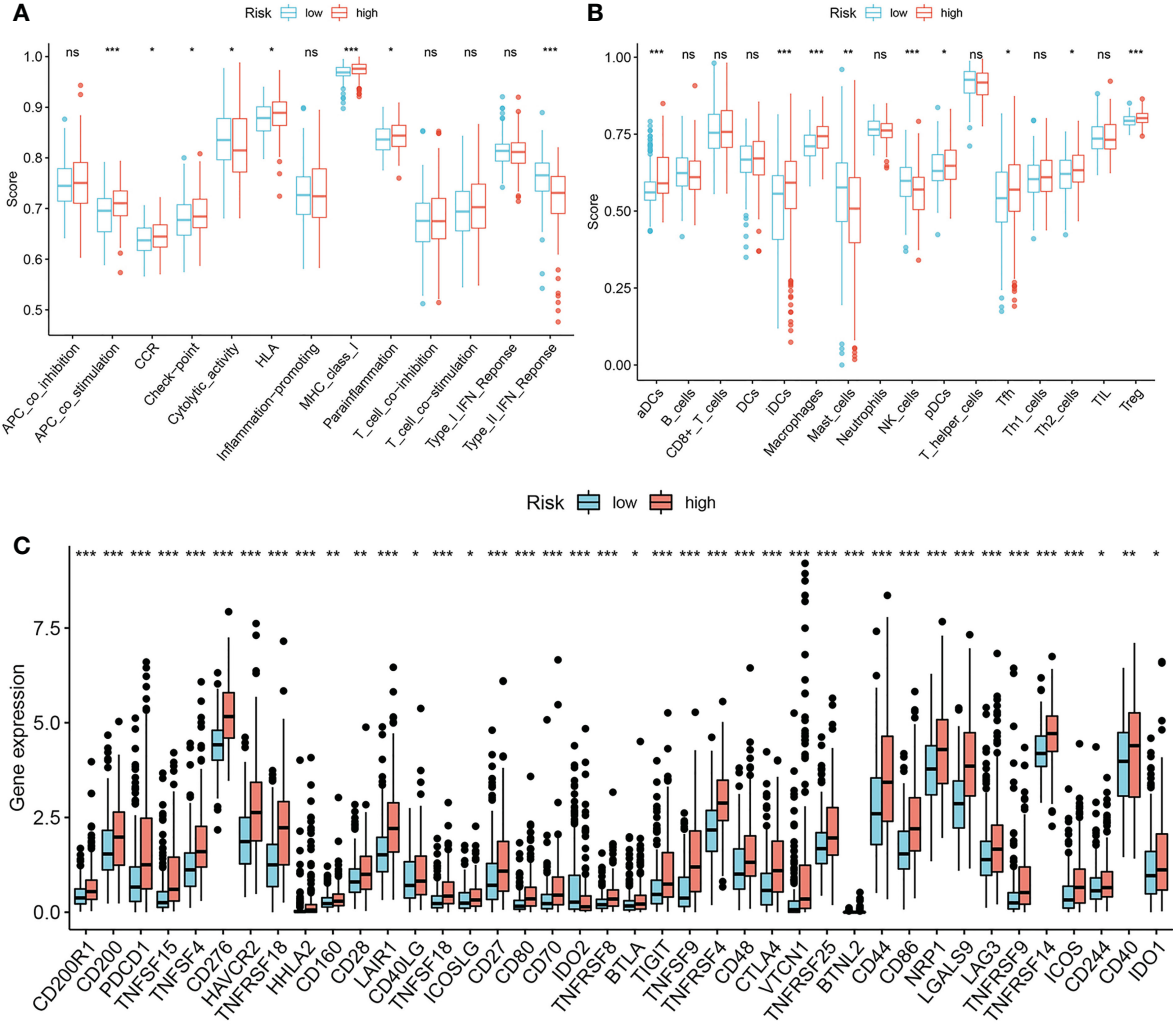
Associations between prognostic signature and immune infiltrating cells/immune checkpoints. **(A, B)** single sample Gene Set Enrichment Analysis. **(C)** Expression pattern of 40 immune checkpoints between the low-risk group and the high-risk group. *P-value < 0.05, **P-value < 0.01 and ***P-value < 0.001, ns, represents non-significant.

The ssGSEA results suggested that high risk score is significantly correlated with more active immune activities and higher infiltration levels of immune cells like APC co-stimulation, CCR, checkpoint, HLA, Para inflammation, MHC class I, aDCs, iDCs, macrophages, pDCs, Tfh, Th2 and Tregs (**Figures 10A, B**). We also determined that high risk score significantly correlates with multiple immune checkpoints, including LAG3, CTLA4, and PD1 (**Figure 10C**).

### 3.8 Distinct mutation characteristics in low-risk group and high-risk group

**Figure 11:**
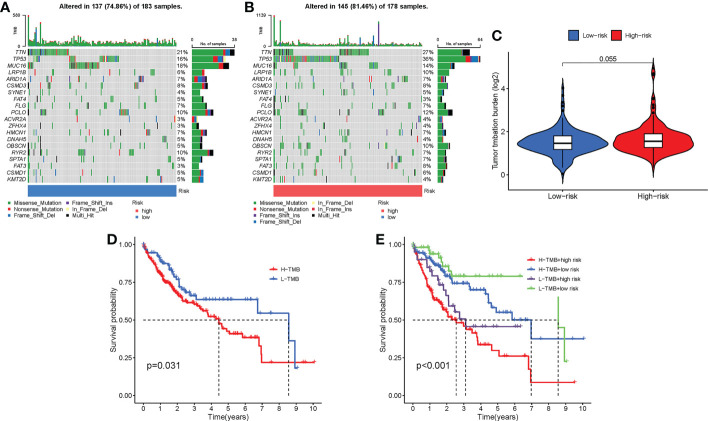
Mutation differences between the low-risk group and the high-risk group. **(A)** Mutation landscape in the low-risk group. **(B)** Mutation landscape in the high-risk group. **(C)** Differences in TMB between the two risk groups. **(D)** Survival analysis between patients with low TMB and patients with high TMB. **(E)** Survival analysis combining risk score and TMB.

We displayed the top 20 most frequently altered genes in the low-risk group and the high-risk group, respectively (**Figures 11A, B**). TTN (21%) and TP53 (36%) were deciphered to be the most frequently altered genes in the low-risk group and the high-risk group, respectively, and the most common mutation type was observed to be missense mutation. We also compared the TMB difference between the two risk groups, which turned out to be not statistically significant (*P* = 0.055) (**Figure 11C**). Patients with high TMB harbor poorer clinical outcomes than those with low TMB (*P* = 0.031) (**Figure 11D**). Survival analysis combining risk score and TMB revealed that patients carrying low TMB and low risk score have the best prognosis, while patients taking high TMB and high-risk score suffered from the worst prognosis (*P* < 0.001) (**Figure 11E**).

### 3.9 Patients in low-risk group were potentially sensitive to several drug agents

**Figure 12:**
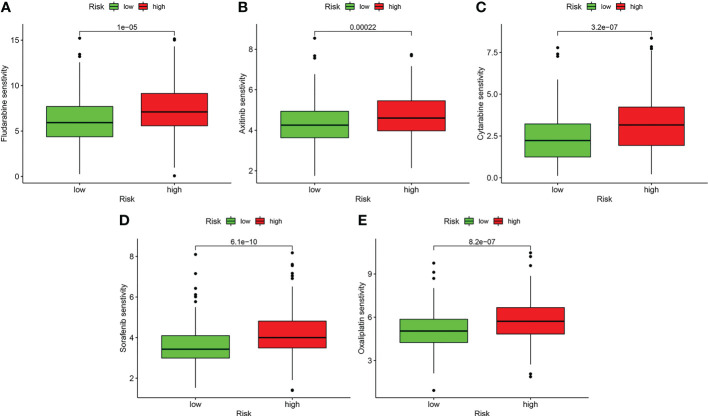
Drug sensitivity analysis. **(A)** Fludarabine. **(B)** Axitinib. **(C)** Cytarabine. **(D)** Sorafenib. **(E)** Oxaliplatin.

Drug sensitivity analysis with IC50 indicated that patients in the low-risk group may more sensitively respond to fludarabine, axitinib, cytarabine, sorafenib, and oxaliplatin (*P* < 0.001) (**Figure 12**).

### 3.10 Single-cell analysis of the M0-related genes

**Figure 13:**
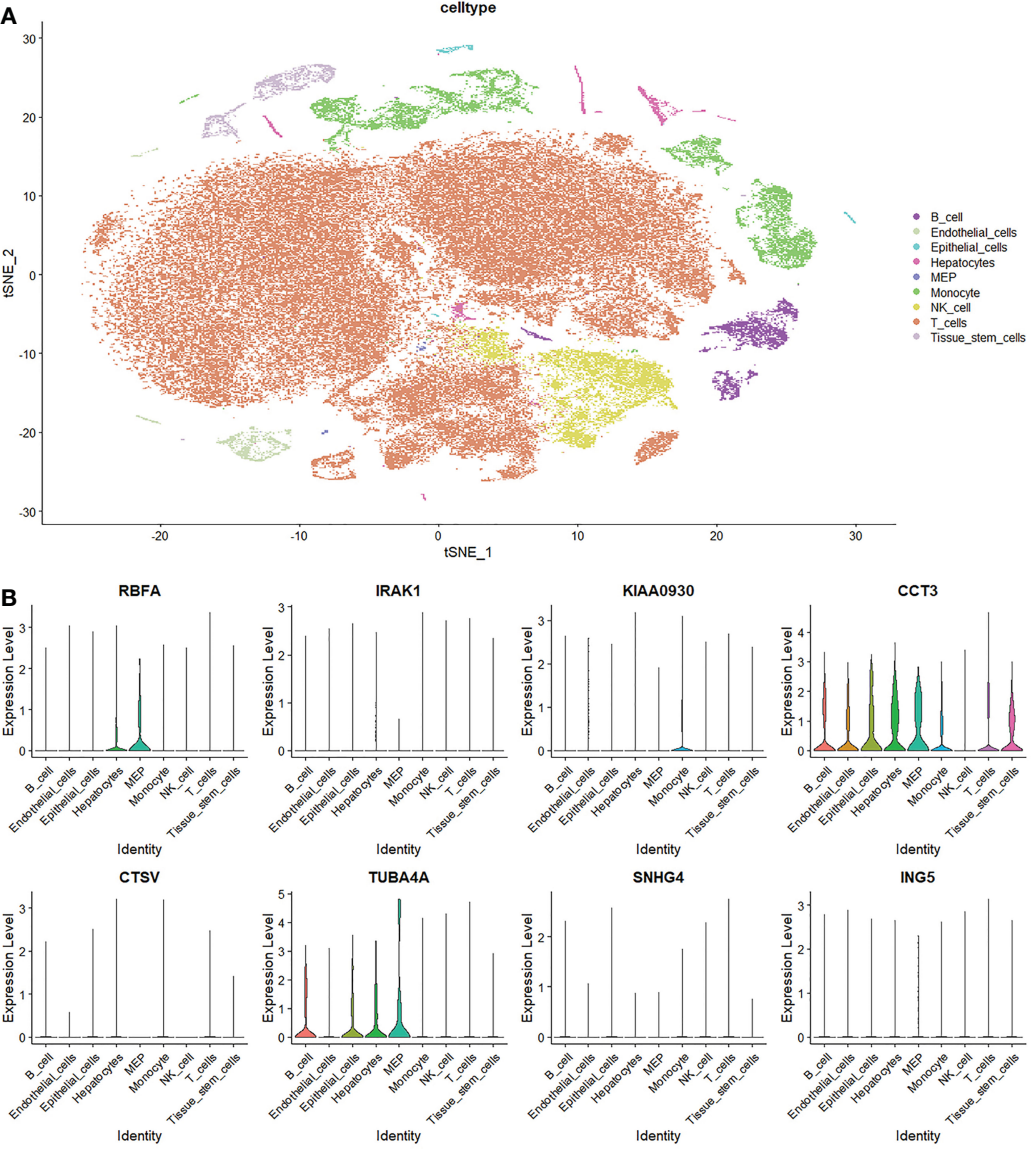
Single-cell analysis of macrophages M0-related genes. **(A)** Annotation of cell subclusters in the tumor microenvironment of LIHC. **(B)** Expression pattern of MRGs in the tumor microenvironment.

To further understand the expression pattern of MRGs in the tumor microenvironment (TME) of LIHC, we processed investigations based on single-cell profiles. It was found that the expression levels of RBFA, KIAA0930, CCT3, and TUBA4A were detected in various cell types in the TME (**Figure 13**). RBFA was detected in hepatocytes and megakaryocyte-erythroid progenitor cells. KIAA0930 was detected in monocytes. CCT3 was detected in B cells, endothelial cells, epithelial cells, hepatocytes, megakaryocyte-erythroid progenitor cells, monocytes, T cells, and tissue stem cells. TUBA4A was detected in B cells, epithelial cells, hepatocytes, and megakaryocyte-erythroid progenitor cells.

## 4 Discussion

Though progress has been made in achieving better survival probability for patients with LIHC, the general prognosis remains unsatisfactory on account of local recurrence and distant metastasis (2, 3). It is getting prevalent to exploit models for prognosis prediction and risk stratification currently. It is worth mentioning that Zhang et al. (17) first report a macrophages M0-related gene model to predict the survival of patients with LIHC. However, our present prognostic signature has several following distinctions and advantages. Firstly, genes that are negatively or positively correlated with macrophages M0 were both included for subsequent analysis. Secondly, we constructed the prognostic signature with ten MRGs, which renders it more robust. What’s more important, the predictive capability of our prognostic signature was stronger, we have higher AUC values in both the training cohort and the validation cohort, which indicated the priority of the present signature to be applied in clinics. In addition, the risk score showed the highest predictive value compared with other traditional clinicopathological features, suggesting the potential advantage of the present signature in aiding practical decision-making. We also applied the prognostic signature in the training cohort, internal validation cohort, and external validation cohort sequentially. Thus, the applicability is verified more rigorously. Subgroup analysis further confirmed the broad applicability of the prognostic signature in all types of patients with LIHC. In addition, the expression pattern of several MRGs in the TME was detected by single-cell analysis. Thus, the macrophages M0-related prognostic signature constructed in the present study may be more clinically practical.

The ten-MRG prognostic signature revealed favorable predictive capability for patients with LIHC, which was more accurate than other clinicopathological factors like grade, T stage, clinical stage, etc. In addition, the risk score was deciphered as an independent prognostic predictor for patients with LIHC, indicating the strong predictive power of the macrophages M0-related gene signature. Macrophages M0 are the undifferentiated cell type that can be potentially induced to polarized cell types, M1 or M2, according to corresponding signals and microenvironment. Macrophages M1 are inflammation-promoting macrophages that secret inflammatory factors, which are caused by lipopolysaccharide (LPS) with or without Th1 cytokines (IFN‐γ, GM‐CSF, etc.). In contrast, macrophages M2 are induced by Th2 cytokines (IL-4, IL-13, etc.) to exert anti-inflammatory and immune-modulatory effects *via* producing anti-inflammatory factors (18, 19). The regulatory role of macrophages M0 in LIHC remains incompletely demonstrated. We noticed that the ten MRGs presented in this study are all risk factors for the prognosis of LIHC. To this extent, our study lies in the primary demonstration of the association between macrophages M0 phenotype and the prognosis of LIHC. However, more experimental evidence is required to strengthen our implication.

Another significance of the present study revealed that several metabolic activities (fatty acid metabolism, bile acid biosynthesis, retinol metabolism, and amino acid metabolism) are significantly upregulated in low-risk group with better prognosis and relatively low macrophages infiltration. Aberrant substance metabolism or metabolic reprogramming is commonly observed in malignancies whereby tumor cells positively respond to metabolic stress caused by glucose deficiency and hypoxia microenvironment (20). The liver is the largest organ that physiologically undertakes the degradation of metabolites and the synthesis of pivotal substances like urea and albumin (21). Thus, the metabolic stress would even be increased during hepatocarcinogenesis. The processing of glucose, fatty acid, amino acid, and glutamine is generally enhanced in liver cancer cells (22). On the other hand, the liver also functions as an immune organ orchestrated by antigen-presenting cells and lymphocytes wandering around the hepatic sinusoids (23). Thus, in the double settings, liver-resident immunocytes attach great importance to metabolic dysregulation in liver diseases. For instance, the switch between polarized macrophages (from M2 to M1) determined the transformation of the inflammatory microenvironment in the progression of obesity (24). But the complex regulatory network behind is largely unexplored, especially that relevant to macrophages. Macrophages in the TME are also named tumor-associated macrophages (TAMs), which are versatile in carcinogenesis (25). A study regarding the TAMs-LIHC interaction ascertained that TAMs could propel the migration of cancer cells by means of stimulating cellular fatty acid oxidation *via* secreting IL-1β (26). Thus, based on our findings, it is suggested that the TAMs may potentially contribute to aberrant substance metabolism like fatty acid oxidation to affect the malignant phenotypes of liver cancer cells. More experimental analyses are necessary to further explore the association between TAMs and metabolic dysregulation in LIHC.

Immunotherapy, as a promising anti-cancer strategy, has somewhat improved the survival probability of patients with LIHC. Massive tumor-infiltrating immune cells resident in the hepatic sinusoids are potential to be activated by stimulation of immune checkpoint blockade (27–29). Investigation of immune checkpoint expression pattern indicated that key immune checkpoints like PD1 and CTLA4 are significantly upregulated in the high-risk group. Thus, immune checkpoint blockade may better benefit patients in the high-risk group, where lies the value of the M0-related prognostic signature in guiding immunotherapy of patients with LIHC. Excessive gene mutations are one of the triggers for tumorigenesis, especially the tumor suppressor genes (30). TTN and TP53 were determined to be the dominant carcinogenesis-driven genes in the low-risk group and the high-risk group respectively, suggesting the possibility of targeting the two dominant genes for prognosis improvement in different risk groups. Several drug agents were also implied by the M0-related prognostic signature to guide the clinical treatment strategy for patients in low-risk group. For instance, the multi-kinase inhibitor sorafenib is originally suitable for patients with unresectable LIHC. Thus, our findings may serve as a clinical reference to apply sorafenib to patients with low risk score. Additionally, the other four drug agents (fludarabine, axitinib, cytarabine, and oxaliplatin) lack the indication in LIHC. Our findings may imply that clinical trials can be conducted to explore the clinical benefits of applying these old drugs in LIHC.

Single-cell transcriptome data is sequenced from annotated cells with high quality, which renders it more precise than common bulk RNA-sequencing data. Thus, it is widely applied to dissect the TME to further understand the intertumoral heterogeneity (31–33). In the present study, we detected the expression pattern of MRGs in the TME based on single-cell analysis. Results revealed that T cells are the most abundant immune infiltrating cells in the TME. Besides, the active expression of two MRGs, CCT3, and TUBA4A, was determined in multiple immunocytes and stromal cells in the TME. Zheng et al. (31) identified 11 T cell subclusters in the TME based on single-cell technology and clinical LIHC samples. They found that the exhausted CD8+ T cells and Tregs were predominant and potentially clonally expanded in the TME. Other studies also indicated the association between exhausted CD8+ T cells infiltration and unfavorable clinical outcomes in LIHC (34, 35). Thus, positive activation of exhausted CD8+ T cells may help to reverse the poor prognosis. In addition, the interaction between TAMs and T cells may be potentially mediated by the two MRGs, CCT3 and TUBA4A, in LIHC, which requires further investigation.

However, there are certain limitations in the present study. Firstly, specimens from actual clinical patients are needed to get precise verification of the expression of the MRGs. Secondly, a prospective study with a large LIHC cohort from multiple centers will make the M0-related prognostic signature and corresponding results more convincing. Thirdly, more experimental studies are required to further unfold the obscure regulatory axes and functional characterizations of the MRGs in LIHC.

## 5 Conclusions

In this present study, a ten-gene prognostic signature was constructed and validated based on macrophages M0-related genes in LIHC. Substance metabolism, like fatty acid metabolism, was significantly strengthened in the low-risk group, which may potentially result from TAMs modulation. Multi-dimensional investigations verified the clinical utility of the prognostic signature. Furthermore, single-cell analysis dissected the active expression of MRGs in the TME of LIHC. Taken together, this macrophages M0-related gene signature may provide new insights into prognostic prediction, risk decision, and clinical treatment strategy for patients with LIHC.

## Data availability statement

The original contributions presented in the study are included in the article/Supplementary Material. Further inquiries can be directed to the corresponding author.

## Author contributions

XX and JW designed the study. XX collected and analyzed the data. XX drafted the initial manuscript. JW reviewed and edited the article. All authors approved the final manuscript. All authors contributed to the article and approved the submitted version.
